# Pharmacogenetics of Cardiovascular Prevention in Diabetes: From Precision Medicine to Identification of Novel Targets

**DOI:** 10.3390/jpm12091402

**Published:** 2022-08-29

**Authors:** Mario Luca Morieri, Caterina Pipino, Alessandro Doria

**Affiliations:** 1 Department of Medicine, University of Padova, 35100 Padua, Italy; 2 Metabolic Disease Unit, University Hospital of Padova, 35100 Padua, Italy; 3 Center for Advanced Studies and Technology (CAST), G. d’Annunzio University, 66100 Chieti, Italy; 4 Department of Medical, Oral and Biotechnological Sciences, G. d’Annunzio University, 66100 Chieti, Italy; 5 Section on Genetics & Epidemiology, Joslin Diabetes Center, Boston, MA 02215, USA; 6 Department of Medicine, Harvard Medical School, Boston, MA 02115, USA

**Keywords:** cardiovascular prevention, diabetes, individualized medicine, single-nucleotide polymorphisms, genome-wide analyses, glycemic control, lipid-lowering treatments

## Abstract

Pharmacogenetics—a branch of precision medicine—holds the promise of becoming a novel tool to reduce the social and healthcare burdens of cardiovascular disease (CVD) and coronary artery disease (CAD) in diabetes. The improvement in cardiovascular risk stratification resulting from adding genetic characteristics to clinical data has moved from the modest results obtained with genetic risk scores based on few genetic variants, to the progressively better performances of polygenic risk scores based on hundreds to millions of variants (CAD-PGRS). Similarly, over the past few years, the number of studies investigating the use of CAD-PGRS to identify different responses to cardio-preventive treatment has progressively increased, yielding striking results for lipid-lowering drugs such as proprotein convertase subtilisin/kexin type 9 (PCSK9) inhibitors and statins. The use of CAD-PGRS to stratify patients based on their likely response to diabetes-specific interventions has been less successful, but promising results have been obtained with regard to specific genetic variants modulating the effects of interventions such as intensive glycemic control and fenofibrate. The finding of diabetes-specific CAD-loci, such as *GLUL*, has also led to the identification of promising new targets that might hopefully result in the development of specific therapies to reduce CVD burden in patients with diabetes. As reported in consensus statements from international diabetes societies, some of these pharmacogenetic approaches are expected to be introduced in clinical practice over the next decade. For this to happen, in addition to continuing to improve and validate these tools, it will be necessary to educate physicians and patients about the opportunities and limits of pharmacogenetics, as summarized in this review.

## 1. Precision Medicine: Definition and Applications

The prevalence of diabetes in adults aged 20–79 years is projected to increase from 10.5% (537 million people) in 2021 to 12.2% (783 million of people) by 2045 [1]. This represents a major global health threat with important social and financial implications [2] given that, despite the overall improvement in the treatment of diabetes and its complications over the past few decades [3,4], patients with diabetes still have shorter life expectancy and greater morbidity than patients without diabetes [5]. This is in large part due to a higher risk of cardiovascular disease (CVD) [4], which is still twice as high as in non-diabetic subjects [3,6], making CVD one of the major causes of morbidity and mortality in this population [4]. Thus, novel approaches to improve the treatment of diabetes and prevention cardiovascular complications are urgently needed.

Pharmacogenetics—a branch of precision medicine—has been proposed as a novel tool to achieve this goal. The main goal of precision medicine is to identify subgroups of subjects sharing similar characteristics that make them optimal candidates for specific preventive and/or therapeutic interventions, thereby improving the cost-effectiveness of treatments. This is achieved by maximizing treatment efficacy (i.e., by identifying subjects deriving the greatest clinical benefit from a treatment) and/or minimizing risk and error (i.e., by identifying subjects who are at increased risk of side effects or who might not require a costly treatment that would be otherwise conventionally prescribed). Pharmacogenetics aims to achieve these goals through the use of genetic markers.

**Figure 1:**
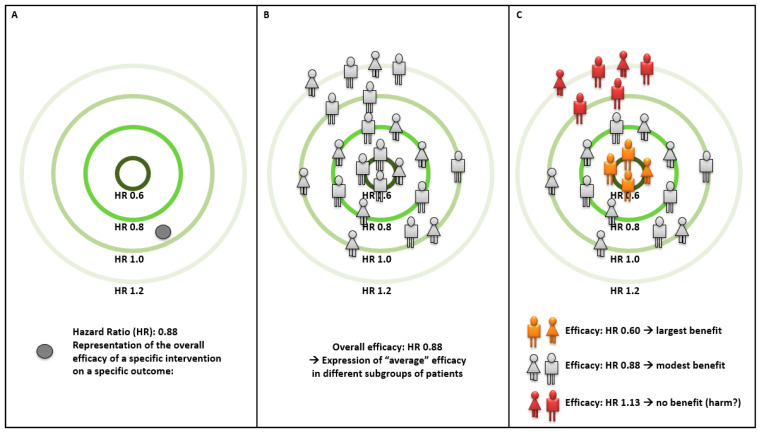
Graphical representation of precision medicine approaches improving clinical benefit of an inter-vention (overall described in panel **A**) through identification of subgroups of subjects (overall de-scribed in Panel **B**) with a larger relative benefit (i.e., smaller hazard ratio) from that treatment (depicted in panel **C**).

Figure 1 shows a schematic representation of a precision medicine approach to improve treatments of patients with diabetes. In panel A, the efficacy of a specific intervention on a specific outcome (e.g., sodium glucose cotransporter-2 inhibitors (SGLT2i) or glucagon-like peptide 1 receptor agonists (GLP1-RA) on major adverse cardiovascular events—MACE [7,8]), as quantified by its hazard ratio (HR), is shown in a target where the center corresponds to the lowest HRs (i.e., largest benefit) and the outer part corresponds to the highest HRs (i.e., no benefit or even a detrimental effect). The overall estimated benefit (in this example, 12% relative risk reduction) is the average of the benefit exerted by the intervention in different subgroups of individuals (Figure 1, panel B). As shown in panel C, the aim of precision medicine is, therefore, to identify characteristics (e.g., a set of genetic variants) that can distinguish those patients at the center of the target (in orange, experiencing the greatest benefit) from those on the edges (in red, experiencing no benefit or potentially being harmed by the treatment).

**Figure 2:**
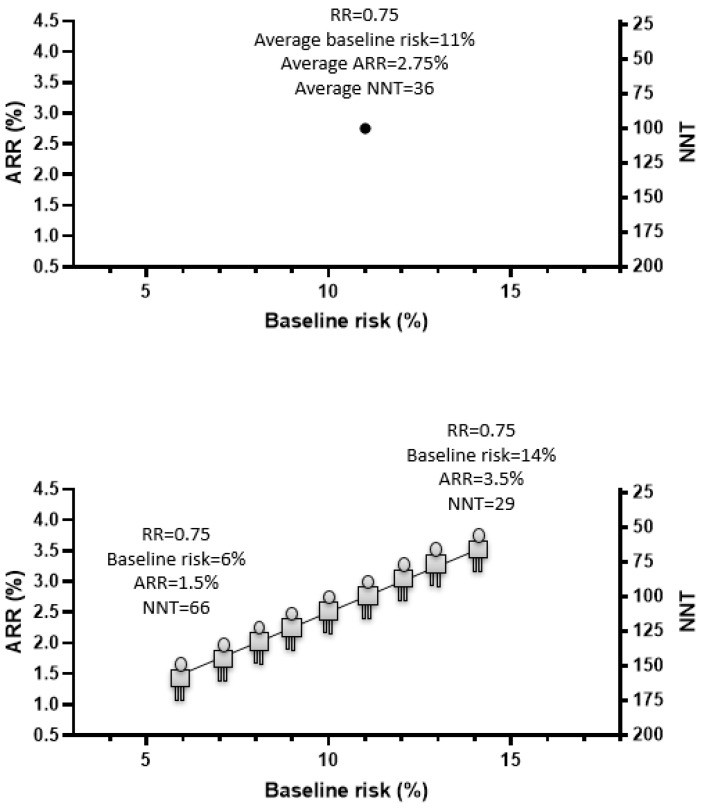
Graphical representation of precision medicine approaches improving clinical benefit of an intervention through identification of subgroup of subjects with a larger baseline risk of the disease of interest.

It is important to note that precision medicine approaches can be applied even in the absence of significant heterogeneity in treatment response, i.e., even if all patients experience the same risk reduction from an intervention. This is accomplished by identifying subjects with a higher baseline risk of disease. If we express the benefit of a clinical intervention as the number of subjects needed to be treated (NNT) to prevent one harmful event (e.g., MACE) over time, this corresponds to the reverse of the absolute risk reduction (ARR) derived from that treatment (NNT = 1/ARR). Since the ARR depends on both the baseline risk of the population and the relative risk reduction (ARR = [baseline risk] − [baseline risk] * [Relative Risk]), this can be increased through the identification of subjects with higher baseline risk even if the relative risk is constant across population subgroups. Figure 2 shows how the average ARR and NNT frequently reported from clinical trials would change if one could identify subjects with different baseline risks of CVD.

Besides being useful for precision medicine purposes, the identification of shared characteristics allowing the selection of patients with different responses to treatments might also lead to the discovery of novel genes or pathways involved in disease etiology or in the mechanism of action of specific interventions, which can then be leveraged for the development of new treatments for diabetes and/or its complications.

As summarized in the consensus report of the Precision Medicine Diabetes Initiative (PMDI—supported by the American Diabetes Association (ADA) and European Association for the Study of Diabetes (EASD) societies), one of the key aspects of precision medicine (as opposed to standard medical approaches) is to integrate multidimensional, complex data to account for individual differences (i.e., individual’s health status, predisposition, prognosis, and likely treatment response) [9]. Therefore, while the focus of this review is on pharmacogenetics, one should consider that several other -omics and non-omics approaches can be used to develop and implement a precision medicine strategy among patients with diabetes.

This review will present some key examples of pharmacogenetics studies of diabetes and cardiovascular complications. First, we will show how genetic variants associated with CVD can improve the identification of diabetic patients at higher risk of CVD who may derive greater benefit from certain cardiovascular preventive strategies (e.g., the use of proprotein convertase subtilisin–kexin type 9-inhibitors (PCSK9i)). We will then describe two examples of pharmacogenetic studies using either a genome-wide approach or a candidate-gene approach that have led to the identification of genetic variants that are characteristic of diabetic patients who experience greater benefits and/or lower risks of adverse events from intensive glycemic control and fenofibrate treatment. Finally, we will show how precision medicine studies aiming to identify genetic variants associated with CAD risk among patients with diabetes can also lead to the discovery of novel pathways linking diabetes to CAD, which can then become targets for the development of novel pharmacological treatments.

## 2. Pharmacogenetic Studies Using Polygenic Risk Score for CAD: CAD-PGRS

### 2.1. Polygenic Risk Score for CAD to Improve CV Risk Assessments

Over the past 15 years, a constantly growing numbers of genome-wide association studies, conducted in consistently larger populations (including thousands or even millions of individuals), have led to a better understanding of the genetic background of CVD [10,11,12]. This has been possible thanks to the advantages of novel genotyping techniques (i.e., next-generation sequencing (NGS)) allowing the parallel sequencing of nucleotides in targeted regions or the entire genome, at progressively lower cost. As of today, more than 200 independent variants have been found to be robustly associated with CAD in the general population. For most of these, the association has been confirmed among subjects with diabetes [13,14,15,16,17], raising the possibility of using this genetic information to improve the risk prediction of CAD/CVD in this population [16,18].

Most of the variants identified to date are common polymorphisms associated with only a small increase in CVD risk (in the order of 5–10 % per risk allele). Therefore, in order to be useful for improving CV risk prediction, several of them must be combined together in a polygenic risk score (PGRS). The usefulness of combining these variants has been demonstrated in the general population [19,20], and we have shown that the same is true among patients with diabetes [16]. Specifically, through the analysis of genome-wide-data from the ACCORD clinical trial, we found that a PGRS combining 204 CAD-associated single-nucleotide polymorphisms (SNPs) identified in the general population was strongly associated with incident major coronary events in patients with diabetes, independent of classic cardiovascular risk factors such as age, gender, smoking habits, lipid and blood pressure control, and personal or family history of CVD. These results were confirmed in the ORIGIN study including subjects with diabetes or pre-diabetes at high or very-high CV risk [16]. Importantly, we found that the PGRS significantly improved CV risk prediction performance when added to a “classic” CV risk score calculator such as the AHA/JACC pooled cohort equation. While the classic CVD risk factors outperformed the PGRS alone in predicting CAD events, the addition of the PGRS to these variables produced a substantial increase in the discrimination of subjects who experienced a CAD event from those who did not, as expressed by a significant increase in the Net Reclassification Index (NRI) and relative integrated discrimination improvement (rIDIs). As reviewed elsewhere [21], improving these indices (even when the increase in c-statistics is marginal) can be very useful to prioritize the allocation of preventive treatments that are especially invasive and/or expensive. As described in the next paragraph, a tangible example of this strategy is provided by two recent post hoc analyses of randomized clinical trials of PCSK9i [22,23].

### 2.2. Polygenic Risk Score for CAD to Improve Allocation of PCSK9i Treatments

**Figure 3:**
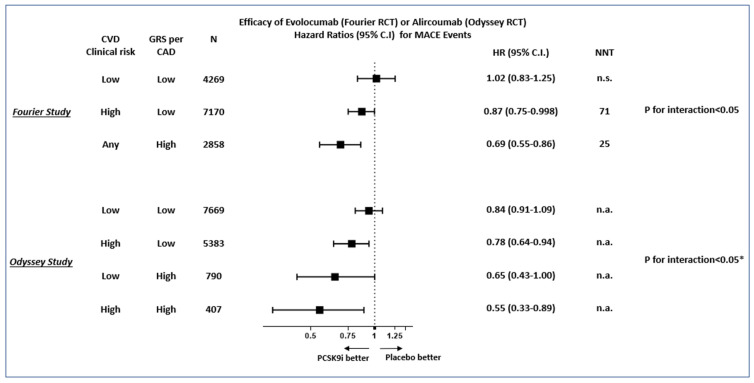
Efficacy of the PCSK9 inhibitors evolocumab and alirocumab in the FOURIER and ODYSSEY studies (patients in secondary cardiovascular prevention) stratified by the combination of clinical and genetic CVD risk information. Notes: in the FOURIER study, the clinical risk score is based on the presence of multiple risk factors, while in the ODYSSEY study this is exclusively on the basis of LDL cholesterol values > or < 100 mg / dl. NNT = number needed to treat over the study observation period (FOURIER 2.3 years) to prevent one MACE event. * In the ODYSSEY study, only the GRS × treatment interaction p was reported as significant, and the effect between efficacy of alirocumab in the low genetic and clinical risk group was significantly less than in the high genetic and clinical risk group (adapted from Damask et al., *Circulation* 2020 [23] and Marston et al. *Circulation* 2020 [22]).

PCSK9i are very effective and well-tolerated LDL cholesterol-reducing drugs with proven cardiovascular efficacy among subjects at very high cardiovascular risk [24,25]. However, they are very expensive and seldom reimbursed by national health systems or healthcare providers [26,27,28], making their cost-effectiveness unclear [29]. One of the strategies that can be pursued to optimize their use is, therefore, to improve the identification of those subjects who could benefit the most from these drugs. In this context, two recent post hoc analyses of RCTs (FOURIER and ODYSSEY studies, including subjects with prior CVD) have shown very promising results using PGRS for CAD. Both studies showed that treatment with PCSK9i caused larger relative and absolute reductions in major CVD event (MACE) risk among subjects with higher PGRS than among those with lower PGRS. As shown in Figure 3, despite the methodological differences, both studies showed that by combining simple clinical and genetic data, one can distinguish subjects who will derive a large benefit from these drugs from those who will experience limited or no benefit (at least in the relatively short duration of the trial, i.e., 2–3 years, and when added to maximum tolerated statin–ezetimibe treatment) [22,23]. It should be noted that the CAD-PGRS used in these studies were different (based on 27 SNPs associated with CAD in FOURIER and based on six million variants in the ODYSSEY), yet they were both characterized by a clear trend for interaction with PCSK9i treatment (reported as significant in FOURIER and estimated to be significant in ODYSSEY). These studies were conducted in large mixed populations including both diabetic and non-diabetic subjects (33% with diabetes among the 14,298 subjects included in FOURIER and 28% with diabetes among the 18,942 subjects included in ODYSSEY) and, therefore, results will need to be validated and replicated in ad hoc studies in subjects with diabetes. Nonetheless, these findings clearly show the potential clinical utility of this approach. Moreover, besides the differences in the relative efficacy of PCSK9i across the GRS strata, one should consider that the patients with larger relative benefit (i.e., with lower HR) because of the high GRS also had a higher baseline risk of CVD. Thus, the differences in NNT across PGRS groups are expected to be even larger.

### 2.3. Polygenic Risk Score for CAD and Allocation of Other Cardiovascular Preventive Treatments

Results similar to those for PCSK9i have been obtained with statins. These studies have shown that (1) subjects with higher CAD-PGRS are characterized by larger statin-induced CVD benefit (both in primary and secondary CVD prevention) [30,31], (2) combining clinical and genetic information can identify subjects at intermediate CV risk who would benefit more from statin treatments [32], and (3) sharing genetic testing results with patients may increase their adherence to treatment [33] and healthy behavioral changes [34]. However, most of these studies were conducted in the general population and only few investigated whether CAD-PGRS could help identify subjects with diabetes experiencing greater benefit from these treatments. In the ACCORD study, the above-mentioned CAD-PGRS based on 204 SNPs, which successfully identified diabetic subjects with higher CAD risk, was not found to influence the effect on CAD of the three randomized interventions being tested in the study (i.e., intensive vs. standard glycemic control, fenofibrate + simvastatin vs. placebo + simvastatin, and intensive vs. standard blood pressure control) [16]. However, while this suggests that the genetic heterogeneity captured by the CAD-PGRS may not generally overlap with the heterogeneity in response to these treatments, individual variants at some of the CAD loci, in particular, those involved in the pathways directly targeted by treatments, may individually influence treatment outcomes. For instance, the common gain of function mutation in the *LPL* (lipoprotein lipase) gene, a well-known CAD locus included in the CAD-PGRS based on 204 SNPs, has been found to influence the cardiovascular effectiveness of fenofibrate [35]—a PPAR-alpha agonist with known effects on LPL expression and lipoprotein lipase activity [36]. Therefore, more specific CAD-PGRS capturing of the biological pathways linking each specific intervention to CAD may be more useful to identify subjects with a better response to treatments. This is discussed in more detail in the next paragraphs describing pharmacogenetic studies of intensive glycemic control and fenofibrate [37,38,39].

More recently, a post hoc analysis of the ADVANCE trial, which tested the CVD effectiveness of intensive blood pressure and glucose control in individuals with T2D, evaluated the usefulness of multi-genetic risk scores, including variants associated with 10 different diabetes-related traits (e.g., blood pressure, glomerular filtration rate, albuminuria, blood pressure, diabetes, and CVD). The authors reported that subjects with a multi-clinical and polygenic risk score (multiPRS, including clinical information and the multi-traits GRS) in the highest 30% of the distribution had a trend for a larger CVD benefit from the combined intensive blood pressure and glucose control (NNT = 12 for CV death over 5 years) as compared to subjects with multiPRS in the lowest 30% (NNT = 66). However, while these differences in NNT are promising, the study did not formally test whether the efficacy in these two groups was statistically different (results of the analysis of interaction were not reported in the paper, and the lack of 95% C.I. did not allow an estimation of this from the NNT scores). In addition, no external replication or validation were reported, prompting great caution in the interpretation of these findings [17].

## 3. Identification of Novel Variants Associated with Different Responses to CV-Preventive Treatments in Diabetes

While the above studies were based on genetic variants identified by their association with increased risk of disease, an alternative approach is to directly search the genome for variants that modulate the effectiveness of cardio-protective treatments. Below, we describe two such pharmacogenetic studies which have provided robust and promising findings, one concerning intensive glycemic control, the other concerning intensive lipid control with fenofibrate [35,37,38].

### 3.1. Pharmacogenetic Studies to Reduce Adverse Effects of Intensive Glycemic Control

As previously discussed, pharmacogenetic studies can also be used to identify subjects at higher risk of detrimental effects of interventions. An example of this is the discovery of genetic variants that may help distinguish subjects who benefit from intensive glycemic control from those who might experience possible harm (e.g., higher risk of cardiovascular death) from this treatment [38]. Indeed, randomized studies have shown that intensive glycemic control, while being associated with a reduction in the risk of MACE and non-fatal myocardial infarction [40] has no benefit or may even have a detrimental effect on cardiovascular mortality [41,42]. For instance, in the ACCORD clinical trial (including more than 10,000 subjects with type 2 diabetes at high or very-high CV risk), intensive glycemic control, i.e., aiming for an HbA1c <6% as compared to 7–8% in the control group, was associated with a significant increase in cardiovascular (+35%) and total (+22%) mortality, which offset the reduction in the risk of non-fatal myocardial infarction (-18%) obtained with this intervention. Based on this evidence, current guidelines do not recommend aiming for an HbA1c value below 6%, despite the known benefit on non-fatal cardiac events and microvascular complications [41].

To identify patients who might benefit from intensive glycemic control without experiencing this increased mortality risk, our group conducted a pharmacogenetic study using data from the ACCORD clinical trial. Through a genome-wide analysis, we identified two different genetic loci (on chromosomes 10 and 5) harboring variants associated with a higher risk of cardiovascular mortality among subjects randomized to intensive glycemic control (i.e., treated to achieve an HbA1c <6%). The leading SNPs at the two loci were associated with 3.6- and 2.7-fold increases per risk allele in the risk of cardiovascular death, respectively (rs9299870, HR: 3.58; 95% C.I. 2.32–5.55 and rs57922, H.R.: 2.65 with 95% C.I. 1.88 to 3.72; *p* values < 5 × 10^−8^ for both) [38]. Importantly, when these two variants were combined in a GRS, the 30% of the population carrying at least two risk alleles had a markedly increased risk of cardiovascular death when treated with intensive glycemic controls without any discernible benefit on non-fatal MI. In contrast, the 70% with zero or 1 risk allele had a benefit of non-fatal MI without any increase in mortality or even with a possible reduction in this adverse outcome. Remarkably, these results were confirmed in an observational setting (the Joslin Kidney Study), in which a similar interaction was observed between the GRS and glycemic control [38]. Moreover, subsequent studies identified a possible decrease in GLP1 levels during intensive glycemic control among carriers of the rs57922 risk allele as a possible mechanism involved in the observed gene by treatment interaction with cardiovascular mortality [39]. Specifically, subjects carrying two risk alleles (T/T homozygotes) had a 28% reduction in GLP-1 levels during intensive glycemic control, whereas C/C homozygotes, i.e., those who derived the maximum cardiovascular benefits from intensive treatment, had an increase in GLP-1 levels during follow-up (with a significant gene-by-intervention interaction). Despite requiring further validation, these findings provide further support to the GWAS results given the known role of GLP1 in CVD and diabetes [7]. They also suggest that T/T homozygotes, in whom intensive glycemic control by traditional means would not be advisable because of the increased mortality and lack of benefit on non-fatal MI, may be good candidates for treatment with GLP-1 agonists—a class of drugs that was not extensively used at the time of the ACCORD trial.

### 3.2. Pharmacogenetic Studies to Expand The Number of Patients That might Benefit from Treatment: The Example of Pharmacogenetic Studies of Fenofibrate

**Figure 4:**
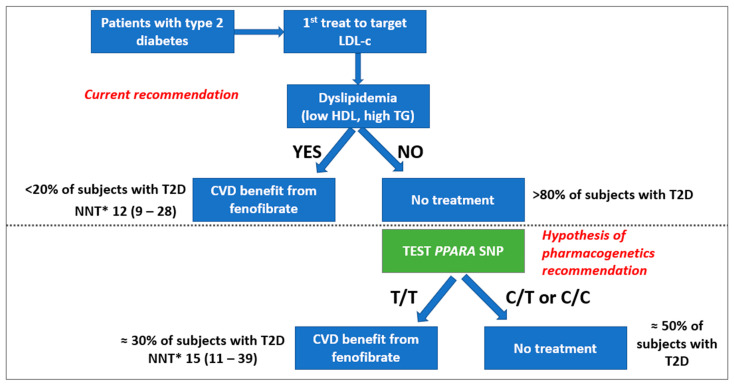
Graphical representation of the hypothesis of pharmacogenetic recommendation to improve the identification of subjects with type 2 diabetes that might have cardiovascular benefit from fenofibrate treatment (* NNT = number needed to treat at 5 years to prevent one MACE, estimated from the population of the ACCORD clinical trial).

Cardiovascular prevention in subjects with diabetes involves lipid-lowering treatments (LLT). Given the established causal role of LDL cholesterol (LDLc) on CVD, the first goal of LLT is to achieve LDLc targets using statins plus ezetimibe and/or PCSK9i when needed [43]. An additional goal is the treatment of “atherogenic dyslipidemia”: a condition characterized by low HDL-c, high triglycerides, and higher levels of atherogenic small-dense LDL particles [44,45]. This condition is important for several reasons: (1) it is more common in patients with diabetes than in the general population; (2) it is associated with higher cardiovascular risk regardless of LDL cholesterol values [46]; and (3) it is amenable to treatment with fibrates (in particular, fenofibrate) or omega-3 fatty acids (e.g., eicosapentaenoic acid) [44,47,48,49,50,51,52]. The last point is of particular relevance for the topic of this review since multiple pre-specified secondary analyses of different RCTs have shown that the presence of atherogenic dyslipidemia can be used to identify those patients with diabetes who are more likely to derive a significant cardiovascular benefit from fenofibrate therapy (i.e., a 35% reduction in relative risk of MACE). Based on this evidence, current clinical guidelines suggest considering fenofibrate treatment in patients with diabetes and atherogenic dyslipidemia once LDL-c targets have been achieved [44,47]. However, as illustrated in Figure 4, less than 20% of diabetic patients have atherogenic dyslipidemia, severely limiting the public health impact of this strategy.

This problem is potentially overcome by the recent identification of a common variant (rs6008845) in the region of *PPARA* (the gene coding for PPAR-alpha—the pharmacological target of fenofibrate), which can be used to identify diabetic patients without atherogenic dyslipidemia who may benefit from fenofibrate as much as those with atherogenic dyslipidemia [37]. In short, from the analysis of ≈3500 subjects in the ACCORD-Lipid study, we found that subjects homozygous for the T allele of this common variant (≈one third of the population) had a 49% reduction in the risk of MACE (95% C.I. 28% to 66%) when randomized to fenofibrate + statin versus placebo + statin. In contrast, subjects carrying either of the other two genotypes did not derive any benefit from this treatment, resulting in a highly significant SNP x fenofibrate interaction (*p* = 0.0004). These findings were replicated in ACCORD participants of African American origin, as well as in prospective observational studies (ACCORD-BP, ORIGIN and TRIUMPH studies) (58). Remarkably, from a clinical standpoint, we found that fenofibrate significantly reduced MACE risk among T/T homozygous even in the absence of dyslipidemia (HR 0.51; 95% C.I. 0.33–0.79) [37]. Therefore, as shown in Figure 4, it may be possible to implement an approach combining clinical and genetic information, according to which one first selects diabetic patients to be treated with fenofibrate based on the presence of atherogenic dyslipidemia—and if this is absent, genotype variant rs6008845—to treat those with the T/T genotype. In support of this strategy, the NNT of fenofibrate among ACCORD participants without dyslipidemia who carried the rs6008845 T/T genotype (≈28% of the total population) was similar to that observed among ACCORD participants with atherogenic dyslipidemia (≈20% of the total population). Thus, through this approach, one could double the number of patients who could benefit from fenofibrate therapy. At the moment, the mechanisms underlying this genetic effect are not completely understood, but they do not seem to involve variability in fenofibrate effects on lipid profile. Further studies are underway to shed light on the pathways that are involved. Nonetheless, even in the absence of a clear explanation of the mechanism of action, this pharmacogenetic approach has the potential to substantially expand the population who can benefit from fenofibrate, thereby increasing the public health relevance of this treatment [53]. Further studies are also warranted to replicate and validate other variants at the *PPARA* locus [37] and other loci (e.g., rs328 on *LPL*) [35] that have been found to influence fenofibrate effectiveness on MACE events in the ACCORD study independently from the SNP rs6008845 (Table 1).

## 4. Searching for Diabetes-Specific CAD Genes to Develop Novel Precision Medicine Treatment

Beyond identifying subgroups of subjects that have a better response to already known cardiovascular interventions, genetic studies can be useful to identify novel targets and treatments for CVD. These new treatments can then be specifically directed to the carriers of the genotype that were originally associated with increased risk of CAD, in a precision medicine fashion. This approach is exemplified by our recent findings suggesting that pharmacological supplementation of the amino acid glutamine might help to prevent CAD in carriers of a genetic variant (SNP rs10911021) [54] that we had previously found to be associated with CAD in multiple populations with type 2 diabetes [55,56,57]. SNP rs10911021 is located at the 1q25 locus near the glutamate–ammonia ligase (*GLUL*) gene, which codes for the enzyme catalyzing the synthesis of glutamine from glutamic acid and ammonia. Patients carrying the risk allele at this locus have a significantly lower expression of GLUL in endothelial cells. They also have a lower plasma pyroglutamic-to-glutamic acid ratio, suggesting an impairment of the γ-glutamyl cycle involved in natural antioxidant glutathione (GSH) production as a possible mechanism through which the SNP confers an increased risk of CAD [55,56,57]. In support of this hypothesis, we found that the risk allele of rs10911021 was associated in endothelial cells with biochemical signs of γ-glutamyl cycle dysfunction and impaired detoxification of the atherogenic compound methylglyoxal (MG)—two traits that may accelerate atherogenesis by intensifying the oxidative stress characteristic of T2D. Consistent with this, GLUL down-regulation by shRNA caused a marked increase in MG levels in endothelial cells. Importantly, the deleterious effects of GLUL down-regulation were completely prevented by exposing cells to glutamine (the product of the enzymatic reaction catalyzed by the enzyme coded by *GLUL*) [52].

Oral administration of pharmaceutical-grade L-glutamine is approved by the Food and Drugs Administration (FDA) to prevent pain crises in patients with sickle cell anemia [58]. This beneficial effect is due to a glutamine-induced increase in the redox ratio ([NADH]:[NAD + + NADH]) in sickle cell erythrocytes, which reduces oxidative stress [59]. One can hypothesize that the same mechanism is involved in the beneficial effect of glutamine observed in endothelial cells carrying the GLUL CAD-risk genotype. Specifically, lower GLUL activity associated with the 1q25 risk allele, which may determine impaired methylglyoxal detoxification and increased oxidative stress in patients with T2D, which can be prevented by the increase in redox ratio provided by glutamine supplementation. Thus, glutamine supplementation could be a simple intervention to decrease CAD risk in T2D patients who carry the 1q25 risk genotype. Even in the absence of this genetic risk factor, the increase in redox ratio induced by glutamine supplementation could be beneficial to those T2D patients who are exposed to excess oxidative stress for other reasons such as uncontrolled hyperglycemia or the presence of other cardiovascular risk factors.

## 5. Advantages and Current Gaps in Using Genetic Variants as Biomarkers for Precision Medicine

As discussed in the previous sections, precision medicine and pharmacogenetic treatment approaches are based on reclassification, i.e., using novel biomarkers to improve the classification of patients into categories with different likelihoods of developing a condition and/or experiencing a beneficial (or detrimental) response to a treatment. In order to implement these approaches in clinical practice, novel biomarkers, such as genetic variants or any other marker used for precision medicine, should be evaluated by answering the following questions [60]: (1) Does the biomarker add novel information? (2) Will the biomarker help the clinician in managing patients? (3) Can the biomarker be easily and reliably measured? As discussed above, there are several examples of genetic markers for CVD in diabetes for which the answer to the first two questions is affirmative. As for the third question, genetic variants have several ideal characteristics: they can be easily measured in blood or saliva samples, their assays are relatively inexpensive and can be carried out in a genome-wide fashion and, most importantly, the information they provide is stable over time, so that they can be measured only once in each patient (e.g., at birth) and provide information on multiple health outcomes throughout life [19].

At the same time, there are several gaps that must be filled in a timely manner in order to accelerate the clinical implementation of pharmacogenetic studies, including those concerning CVD in diabetes. First, it is necessary to increase the quality of published precision medicine studies by validation or external replication of the main findings or a clear discussion of the limits of unconfirmed results. This will increase the likelihood of identifying robust candidates for precision medicine that can be trusted by the medical community, as well as by the lay public. Second, the medical and scientific communities should discuss the type of evidence that is needed to implement pharmacogenetic findings in clinical practice without unnecessary delays. While specific RCTs are certainly needed for any novel therapy, in the case of already approved treatments with established safety profiles (e.g., fenofibrate or PCSK9i), validation in phase 4 studies (ideally with a prospective design) using large biobank datasets, or multiple concordant post hoc analyses of RCTs, might be sufficient for the clinical implementation of pharmacogenetic approaches. At the same time, national and international professional societies, drug companies, and other stakeholders should collaborate in designing novel pharmacogenetic studies whenever data that can be used for pharmacogenetics analyses are not available from biobanks or RCTs.

Third, while pharmacogenetic-guided treatments have already been projected to be cost-effective or even cost-saving in several instances [61], it will be important to conduct such evaluations on a case by case basis.

Finally, an important gap that must be filled concerns the inadequate representation of populations of non-European ancestry in the precision medicine studies that have been conducted to date, which severely limits the generalizability of findings. For instance, the CAD-PGRS based on variants mainly discovered in non-Hispanic white subjects performs poorly in other racial groups, both in the general population [12] and among subjects with diabetes [14,16]. Thus, developing GRSs including ancestry-specific loci and variants [12] is a top priority, as clearly indicated by the ability of multi-ancestry approaches to discover additional genetic variants associated with cardiometabolic disorders [62]. As an example of this, it has been estimated that individuals of African origin (mostly African Americans) represent only 2.4% of the participants in large genetic studies, yet they account for 7% of the disease-related genetic associations being identified by those studies [63,64]. It is also important to consider that some non-European populations are those in which the prevalence of diabetes is expected to rise the most over the next decades. For instance, in Africa, the number of adults with diabetes is expected to rise by 134% from 2021 to 2045, as compared to 13% and 24% increases in Europe and North America, respectively [1]. For this reason, it is crucial to develop country- or population-specific genetic programs over the next few years [64].

## 6. Conclusions

The body of evidence in support of pharmacogenetic approaches to improve the cardiovascular management of patients with diabetes is increasing rapidly, offering hope for more rational and cost-effective treatments of this major health problem. However, the impact of these new approaches is yet to be seen in clinical practice. According to the timeline of the Precision Medicine in Diabetes Initiative formulated by the ADA and the EASD [9], we are currently in “Phase 2”, which corresponds to requests for applications to address gaps, funding of precision medicine research projects, and complete systematic reviews of evidence. “Phase 3”, which includes ongoing research, the dissemination of findings, and the development of clinical guidelines, is expected to be completed by 2025, and the last phase, consisting of the education of physicians and patients, is expected to take place immediately after that. If all appropriate efforts are made to adhere to this timeline, we may soon be able to apply precision medicine approaches to the treatment of patients with diabetes and fully enjoy the benefits of this new strategy.

## Figures and Tables

**Table 1:** Characteristics of the top SNPs at the *PPARA* locus modulating fenofibrate effectiveness in ACCORD among self-reported non-Hispanic Whites and African Americans and in a meta-analysis of the two groups.

SNP	Allele 1/2	White			African American			Meta-Analysis *p* Value	rs6008845 Conditional Analysis *p* Value
		*p* Value	Effect ± S.E.	Allele1 Freq	*p* Value	Effect ± S.E.	Allele1 Freq		
rs6008845	c/t	3.7 × 10^−4^	0.59 ± 0.17	0.40	2.4 × 10^−2^	1.39 ± 0.62	0.79	5.6 × 10^−5^	ref
rs135557	g/a	9.3 × 10^−4^	0.54 ± 0.16	0.44	6.8 × 10^−3^	1.43 ± 0.53	0.72	7.3 × 10^−5^	3.0 × 10^−1^
rs135570	g/a	9.0 × 10^−4^	0.54 ± 0.16	0.46	5.3 × 10^−3^	2.16 ± 0.77	0.83	1.3 × 10^−4^	5.4 × 10^−1^
rs6007904	g/a	6.4 × 10^−4^	0.56 ± 0.16	0.42	1.9 × 10^−1^	0.71 ± 0.54	0.73	2.6 × 10^−4^	9.4 × 10^−1^
rs2105914	g/a	1.9 × 10^−3^	0.50 ± 0.16	0.48	2.7 × 10^−2^	1.74 ± 0.79	0.83	5.0 × 10^−4^	6.8 × 10^−1^
rs135577	a/g	2.2 × 10^−3^	0.56 ± 0.18	0.28	1.1 × 10^−1^	0.70 ± 0.44	0.57	5.8 × 10^−4^	6.4 × 10^−1^
rs11090910	c/t	8.1 × 10^−3^	0.49 ± 0.18	0.27	3.5 × 10^−2^	0.89 ± 0.42	0.41	1.1 × 10^−3^	4.2 × 10^−2^
rs9615904	c/t	1.2 × 10^−2^	0.46 ± 0.18	0.29	2.9 × 10^−2^	1.15 ± 0.52	0.81	2.1 × 10^−3^	1.3 × 10^−2^
rs4508712	g/a	1.6 × 10^−2^	0.43 ± 0.18	0.29	2.1 × 10^−2^	1.18 ± 0.51	0.81	2.4 × 10^−3^	1.2 × 10^−2^
rs9615264	a/g	2.6 × 10^−3^	0.95 ± 0.32	0.09	9.6 × 10^−1^	0.08 ± 1.59	0.02	3.1 × 10^−3^	2.3 × 10^−3^

Legend: Conditional p values include the rs6008845 by fenofibrate interaction term in the models. Effect = beta for SNP by fenofibrate interaction; SE = standard error of the beta. Only SNP passing the study-wide significant threshold of *p* = 6 × 10^−4^ in the meta-analyses or with nominally significant conditional *p* values are shown (adapted from Morieri et al., Diabetes, 2020 [37]).

## Literature Review Section

References for this review were identified through searches of PubMed, Web of sciences and Scopus for articles published from January 1975, to December 2021, by use of the terms “Precision Medicine”, “Pharmacogenetics”, “Pharmacogenomics”, “Genetic variants”, “Personalized medicine”, “SNP” and “GWAS” in combination with the term “Diabetes” and “Cardiovascular disease”. Articles resulting from these searches and relevant references cited in those articles were reviewed. Only articles published in English were included.

## References

[1] Sun H., Saeedi P., Karuranga S., Pinkepank M., Ogurtsova K., Duncan B.B., Stein C., Basit A., Chan J.C., Mbanya J.C. (2021). IDF Diabetes Atlas: Global, regional and country-level diabetes prevalence estimates for 2021 and projections for 2045. Diabetes Res. Clin. Pract..

[2] Saeedi P., Petersohn I., Salpea P., Malanda B., Karuranga S., Unwin N., Colagiuri S., Guariguata L., Motala A.A., Ogurtsova K. (2019). Global and regional diabetes prevalence estimates for 2019 and projections for 2030 and 2045: Results from the International Diabetes Federation Diabetes Atlas, 9(th) edition. Diabetes Res. Clin. Pract..

[3] Rawshani A., Rawshani A., Franzén S., Eliasson B., Svensson A.-M., Miftaraj M., McGuire D.K., Sattar N., Rosengren A., Gudbjörnsdottir S. (2017). Mortality and Cardiovascular Disease in Type 1 and Type 2 Diabetes. N. Engl. J. Med..

[4] Pearson-Stuttard J., Bennett J., Cheng Y.J., Vamos E.P., Cross A.J., Ezzati M., Gregg E.W. (2021). Trends in predominant causes of death in individuals with and without diabetes in England from 2001 to 2018: An epidemiological analysis of linked primary care records. Lancet Diabetes Endocrinol..

[5] Heald A.H., Stedman M., Davies M., Livingston M., Alshames R., Lunt M., Rayman G., Gadsby R. (2020). Estimating life years lost to diabetes: Outcomes from analysis of National Diabetes Audit and Office of National Statistics data. Cardiovasc. Endocrinol. Metab..

[6] Wright A.K., Suarez-Ortegon M.F., Read S.H., Kontopantelis E., Buchan I., Emsley R., Sattar N., Ashcroft D.M., Wild S.H., Rutter M.K. (2020). Risk Factor Control and Cardiovascular Event Risk in People With Type 2 Diabetes in Primary and Secondary Prevention Settings. Circulation.

[7] Sattar N., Lee M.M.Y., Kristensen S.L., Branch K.R.H., Del Prato S., Khurmi N.S., Lam C.S.P., Lopes R.D., McMurray J.J.V., Pratley R.E. (2021). Cardiovascular, mortality, and kidney outcomes with GLP-1 receptor agonists in patients with type 2 diabetes: A systematic review and meta-analysis of randomised trials. Lancet Diabetes Endocrinol..

[8] McGuire D.K., Shih W.J., Cosentino F., Charbonnel B., Cherney D.Z.I., Dagogo-Jack S., Pratley R., Greenberg M., Wang S., Huyck S. (2021). Association of SGLT2 Inhibitors With Cardiovascular and Kidney Outcomes in Patients With Type 2 Diabetes: A Meta-analysis. JAMA Cardiol..

[9] Chung W.K., Erion K., Florez J.C., Hattersley A.T., Hivert M.-F., Lee C.G., McCarthy M.I., Nolan J.J., Norris J.M., Pearson E.R. (2020). Precision Medicine in Diabetes: A Consensus Report From the American Diabetes Association (ADA) and the European Association for the Study of Diabetes (EASD). Diabetes Care.

[10] Hartiala J., Schwartzman W.S., Gabbay J., Ghazalpour A., Bennett B.J., Allayee H. (2017). The Genetic Architecture of Coronary Artery Disease: Current Knowledge and Future Opportunities. Curr. Atheroscler. Rep..

[11] Bodmer W., Bonilla C. (2008). Common and rare variants in multifactorial susceptibility to common diseases. Nat. Genet..

[12] Musunuru K., Kathiresan S. (2019). Genetics of Common, Complex Coronary Artery Disease. Cell.

[13] Qi L., Parast L., Cai T., Powers C., Gervino E.V., Hauser T.H., Hu F.B., Doria A. (2011). Genetic susceptibility to coronary heart disease in type 2 diabetes: 3 independent studies. J. Am. Coll. Cardiol..

[14] Look ARG (2015). Prospective association of a genetic risk score and lifestyle intervention with cardiovascular morbidity and mortality among individuals with type 2 diabetes: The Look AHEAD randomised controlled trial. Diabetologia.

[15] Raffield L.M., Cox A.J., Carr J.J., Freedman B.I., Hicks P.J., Langefeld C.D., Hsu F.-C., Bowden D.W. (2015). Analysis of a cardiovascular disease genetic risk score in the Diabetes Heart Study. Acta Diabetol..

[16] Morieri M.L., Gao H., Pigeyre M., Shah H.S., Sjaarda J., Mendonca C., Hastings T., Buranasupkajorn P., Motsinger-Reif A.A., Rotroff D.M. (2018). Genetic Tools for Coronary Risk Assessment in Type 2 Diabetes: A Cohort Study From the ACCORD Clinical Trial. Diabetes Care.

[17] Tremblay J., Haloui M., Attaoua R., Tahir R., Hishmih C., Harvey F., Marois-Blanchet F.-C., Long C., Simon P., Santucci L. (2021). Polygenic risk scores predict diabetes complications and their response to intensive blood pressure and glucose control. Diabetologia.

[18] Levin M.G., Rader D.J. (2020). Polygenic Risk Scores and Coronary Artery Disease: Ready for Prime Time?. Circulation.

[19] Khera A.V., Chaffin M., Aragam K.G., Haas M.E., Roselli C., Choi S.H., Natarajan P., Lander E.S., Lubitz S.A., Ellinor P.T. (2018). Genome-wide polygenic scores for common diseases identify individuals with risk equivalent to monogenic mutations. Nat. Genet..

[20] Khera A.V., Chaffin M., Zekavat S., Collins R.L., Roselli C., Natarajan P., Lichtman J.H., D’Onofrio G., Mattera J.A., Dreyer R.P. (2019). Whole-Genome Sequencing to Characterize Monogenic and Polygenic Contributions in Patients Hospitalized with Early-Onset Myocardial Infarction. Circulation.

[21] Torkamani A., Wineinger N.E., Topol E.J. (2018). The personal and clinical utility of polygenic risk scores. Nat. Rev. Genet..

[22] Marston N.A., Kamanu F., Nordio F., Gurmu Y., Roselli C., Sever P.S., Pedersen T.R., Keech A.C., Wang H., Pineda A.L. (2020). Predicting Benefit From Evolocumab Therapy in Patients With Atherosclerotic Disease Using a Genetic Risk Score: Results From the FOURIER Trial. Circulation.

[23] Damask A., Steg P.G., Schwartz G.G., Szarek M., Hagström E., Badimon L., Chapman M.J., Boileau C., Tsimikas S., Ginsberg H.N. (2020). Patients With High Genome-Wide Polygenic Risk Scores for Coronary Artery Disease May Receive Greater Clinical Benefit From Alirocumab Treatment in the ODYSSEY OUTCOMES Trial. Circulation.

[24] Steg P.G., Szarek M., Bhatt D.L., Bittner V.A., Brégeault M.-F., Dalby A.J., Diaz R., Edelberg J.M., Goodman S.G., Hanotin C. (2019). Effect of Alirocumab on Mortality After Acute Coronary Syndromes. Circulation.

[25] Schwartz G.G., Steg P.G., Szarek M., Bhatt D.L., Bittner V.A., Diaz R., Edelberg J.M., Goodman S.G., Hanotin C., Harrington R.A. (2018). Alirocumab and Cardiovascular Outcomes after Acute Coronary Syndrome. N. Engl. J. Med..

[26] Arca M., Ansell D., Averna M., Fanelli F., Gorcyca K., Iorga R., Maggioni A.P., Paizis G., Tomic R., Catapano A.L. (2018). Statin utilization and lipid goal attainment in high or very-high cardiovascular risk patients: Insights from Italian general practice. Atherosclerosis.

[27] Morieri M.L., Avogaro A., Fadini G.P. (2020). Cholesterol lowering therapies and achievement of targets for primary and secondary cardiovascular prevention in type 2 diabetes: Unmet needs in a large population of outpatients at specialist clinics. Cardiovasc. Diabetol..

[28] Ray K.K., Molemans B., Schoonen W.M., Giovas P., Bray S., Kiru G., Murphy J., Banach M., De Servi S., Gaita D. (2020). EU-Wide Cross-Sectional Observational Study of Lipid-Modifying Therapy Use in Secondary and Primary Care: The DA VINCI study. Eur. J. Prev. Cardiol..

[29] Abushanab D., Al-Badriyeh D., Marquina C., Bailey C., Jaam M., Liew D., Ademi Z. (2022). A Systematic Review of Cost-Effectiveness of Non-Statin Lipid-Lowering Drugs for Primary and Secondary Prevention of Cardiovascular Disease in Patients with Type 2 Diabetes Mellitus. Curr. Probl. Cardiol..

[30] Natarajan P., Young R., Stitziel N.O., Padmanabhan S., Baber U., Mehran R., Sartori S., Fuster V., Reilly D.F., Butterworth A. (2017). Polygenic Risk Score Identifies Subgroup With Higher Burden of Atherosclerosis and Greater Relative Benefit From Statin Therapy in the Primary Prevention Setting. Circulation.

[31] Mega J.L., Stitziel N.O., Smith J.G., Chasman D.I., Caulfield M., Devlin J.J., Nordio F., Hyde C.L., Cannon C.P., Sacks F.M. (2015). Genetic risk, coronary heart disease events, and the clinical benefit of statin therapy: An analysis of primary and secondary prevention trials. Lancet.

[32] Tikkanen E., Havulinna A.S., Palotie A., Salomaa V., Ripatti S. (2013). Genetic risk prediction and a 2-stage risk screening strategy for coronary heart disease. Arter. Thromb. Vasc. Biol..

[33] Kullo I.J., Jouni H., Austin E.E., Brown S.-A., Kruisselbrink T.M., Isseh I.N., Haddad R.A., Marroush T.S., Shameer K., Olson J.E. (2016). Incorporating a Genetic Risk Score Into Coronary Heart Disease Risk Estimates: Effect on Low-Density Lipoprotein Cholesterol Levels (the MI-GENES Clinical Trial). Circulation.

[34] Christensen K.D., Schonman E.F., Robinson J.O., Roberts J.S., Diamond P.M., Lee K.B., Green R.C., McGuire A.L. (2021). Behavioral and psychological impact of genome sequencing: A pilot randomized trial of primary care and cardiology patients. NPJ Genom. Med..

[35] Morieri M.L., Shah H., Doria A., the Action to Control Cardiovascular Risk in Diabetes (ACCORD) Genetic Study Group (2016). Variants in ANGPTL4 and the Risk of Coronary Artery Disease. N. Engl. J. Med..

[36] Duval C., Muller M., Kersten S. (2007). PPARalpha and dyslipidemia. Biochim. Et Biophys. Acta.

[37] Morieri M.L., Shah H.S., Sjaarda J., Lenzini P.A., Campbell H., Motsinger-Reif A.A., Gao H., Lovato L., Prudente S., Pandolfi A. (2020). PPARA Polymorphism Influences the Cardiovascular Benefit of Fenofibrate in Type 2 Diabetes: Findings From ACCORD-Lipid. Diabetes.

[38] Shah H.S., Gao H., Morieri M.L., Skupien J., Marvel S., Paré G., Mannino G.C., Buranasupkajorn P., Mendonca C., Hastings T. (2016). Genetic Predictors of Cardiovascular Mortality During Intensive Glycemic Control in Type 2 Diabetes: Findings From the ACCORD Clinical Trial. Diabetes Care.

[39] Shah H.S., Morieri M.L., Marcovina S.M., Sigal R.J., Gerstein H.C., Wagner M.J., Motsinger-Reif A.A., Buse J.B., Kraft P., Mychaleckyj J.C. (2018). Modulation of GLP-1 Levels by a Genetic Variant That Regulates the Cardiovascular Effects of Intensive Glycemic Control in ACCORD. Diabetes Care.

[40] Ray K.K., Seshasai S.R.K., Wijesuriya S., Sivakumaran R., Nethercott S., Preiss D., Erqou S., Sattar N. (2009). Effect of intensive control of glucose on cardiovascular outcomes and death in patients with diabetes mellitus: A meta-analysis of randomised controlled trials. Lancet.

[41] Skyler J.S., Bergenstal R., Bonow R.O., Buse J., Deedwania P., Gale E.A., Howard B.V., Kirkman M.S., Kosiborod M., Reaven P. (2009). Intensive glycemic control and the prevention of cardiovascular events: Implications of the ACCORD, ADVANCE, and VA diabetes trials: A position statement of the American Diabetes Association and a scientific statement of the American College of Cardiology Foundation and the American Heart Association. Circulation.

[42] Gerstein H.C., Miller M.E., Byington R.P., Goff D.C., Bigger J.T., Buse J.B., Cushman W.C., Genuth S., Ismail-Beigi F., Action to Control Cardiovascular Risk in Diabetes Study Group (2008). Effects of intensive glucose lowering in type 2 diabetes. N. Engl. J. Med..

[43] Mach F., Baigent C., Catapano A.L., Koskinas K.C., Casula M., Badimon L., Chapman M.J., De Backer G.G., Delgado V., Ference B.A. (2020). 2019 ESC/EAS Guidelines for the management of dyslipidaemias: Lipid modification to reduce cardiovascular risk. Eur. Heart J..

[44] Ferrari R., Aguiar C., Alegria E., Bonadonna R.C., Cosentino F., Elisaf M., Farnier M., Ferrieres J., Filardi P.P., Hancu N. (2016). Current practice in identifying and treating cardiovascular risk, with a focus on residual risk associated with atherogenic dyslipidaemia. Eur. Heart J. Suppl..

[45] Taskinen M.R., Boren J. (2015). New insights into the pathophysiology of dyslipidemia in type 2 diabetes. Atherosclerosis.

[46] Ginsberg H.N., Packard C.J., Chapman M.J., Borén J., Aguilar-Salinas C.A., Averna M., Ference B.A., Gaudet D., Hegele R.A., Kersten S. (2021). Triglyceride-rich lipoproteins and their remnants: Metabolic insights, role in atherosclerotic cardiovascular disease, and emerging therapeutic strategies-a consensus statement from the European Atherosclerosis Society. Eur. Heart J..

[47] American Diabetes Association 10 (2021). Cardiovascular Disease and Risk Management: Standards of Medical Care in Diabetes-2021. Diabetes Care.

[48] Jun M., Foote C., Lv J., Neal B., Patel A., Nicholls S.J., Grobbee D.E., Cass A., Chalmers J., Perkovic V. (2010). Effects of fibrates on cardiovascular outcomes: A systematic review and meta-analysis. Lancet.

[49] Sacks F.M., Carey V.J., Fruchart J.C. (2010). Combination lipid therapy in type 2 diabetes. N. Engl. J. Med..

[50] Bruckert E., Labreuche J., Deplanque D., Touboul P.J., Amarenco P. (2011). Fibrates effect on cardiovascular risk is greater in patients with high triglyceride levels or atherogenic dyslipidemia profile: A systematic review and meta-analysis. J. Cardiovasc. Pharmacol..

[51] Kim N.H., Han K.H., Choi J., Lee J., Kim S.G. (2019). Use of fenofibrate on cardiovascular outcomes in statin users with metabolic syndrome: Propensity matched cohort study. BMJ.

[52] Bhatt D.L., Steg P.G., Miller M., Brinton E.A., Jacobson T.A., Ketchum S.B., Doyle R.T., Juliano R.A., Jiao L., Granowitz C. (2019). Cardiovascular Risk Reduction with Icosapent Ethyl for Hypertriglyceridemia. N. Engl. J. Med..

[53] Morieri M.L. (2022). Heart Failure Burden in Diabetes: Can Fenofibrate Provide Additional Hope?. Diabetes Care.

[54] Pipino C., Shah H., Prudente S., Di Pietro N., Zeng L., Park K., Trischitta V., Pennathur S., Pandolfi A., Doria A. (2020). Association of the 1q25 Diabetes-Specific Coronary Heart Disease Locus With Alterations of the gamma-Glutamyl Cycle and Increased Methylglyoxal Levels in Endothelial Cells. Diabetes.

[55] Qi L., Qi Q., Prudente S., Mendonca C., Andreozzi F., Di Pietro N., Sturma M., Novelli V., Mannino G.C., Formoso G. (2013). Association between a genetic variant related to glutamic acid metabolism and coronary heart disease in individuals with type 2 diabetes. JAMA.

[56] Look ARG (2016). Prospective Association of GLUL rs10911021 with Cardiovascular Morbidity and Mortality Among Individuals With Type 2 Diabetes: The Look AHEAD Study. Diabetes.

[57] Prudente S., Shah H., Bailetti D., Pezzolesi M., Buranasupkajorn P., Mercuri L., Mendonca C., De Cosmo S., Niewczas M., Trischitta V. (2015). Genetic Variant at the GLUL Locus Predicts All-Cause Mortality in Patients with Type 2 Diabetes. Diabetes.

[58] Niihara Y., Miller S.T., Kanter J., Lanzkron S., Smith W.R., Hsu L.L., Gordeuk V.R., Viswanathan K., Sarnaik S., Osunkwo I. (2018). A Phase 3 Trial of l-Glutamine in Sickle Cell Disease. N. Engl. J. Med..

[59] Niihara Y., Zerez C.R., Akiyama D.S., Tanaka K.R. (1998). Oral L-glutamine therapy for sickle cell anemia: I. Subjective clinical improvement and favorable change in red cell NAD redox potential. Am. J. Hematol..

[60] Morrow D.A., de Lemos J.A. (2007). Benchmarks for the assessment of novel cardiovascular biomarkers. Circulation.

[61] Verbelen M., Weale M.E., Lewis C.M. (2017). Cost-effectiveness of pharmacogenetic-guided treatment: Are we there yet?. Pharm. J..

[62] Vujkovic M., Keaton J.M., Lynch J.A., Miller D.R., Zhou J., Tcheandjieu C., Huffman J.E., Assimes T.L., Lorenz K., Zhu X. (2020). Discovery of 318 new risk loci for type 2 diabetes and related vascular outcomes among 1.4 million participants in a multi-ancestry meta-analysis. Nat. Genet..

[63] Gurdasani D., Barroso I., Zeggini E., Sandhu M.S. (2019). Genomics of disease risk in globally diverse populations. Nat. Rev. Genet..

[64] Pereira L., Mutesa L., Tindana P., Ramsay M. (2021). African genetic diversity and adaptation inform a precision medicine agenda. Nat. Rev. Genet..

